# Wait-and-scan management in sporadic Koos grade 4 vestibular schwannomas: A longitudinal volumetric study

**DOI:** 10.1093/noajnl/vdad144

**Published:** 2023-11-03

**Authors:** Sammy M Schouten, Stefan Cornelissen, Patrick P H J Langenhuizen, Thijs T G Jansen, Jef J S Mulder, Jolanda Derks, Jeroen B Verheul, Henricus P M Kunst

**Affiliations:** Department of Otolaryngology, Radboud University Medical Center, Nijmegen, The Netherlands; Department of Otolaryngology, Maastricht University Medical Center+, Maastricht, The Netherlands; Dutch Academic Alliance Skull Base Pathology Radboudumc/MUMC+, Nijmegen and Maastricht, The Netherlands; Gamma Knife Center, Department of Neurosurgery, Elisabeth-TweeSteden Hospital, Tilburg, The Netherlands; Gamma Knife Center, Department of Neurosurgery, Elisabeth-TweeSteden Hospital, Tilburg, The Netherlands; Eindhoven University of Technology, Eindhoven, The Netherlands; Gamma Knife Center, Department of Neurosurgery, Elisabeth-TweeSteden Hospital, Tilburg, The Netherlands; Eindhoven University of Technology, Eindhoven, The Netherlands; Department of Otolaryngology, Radboud University Medical Center, Nijmegen, The Netherlands; Dutch Academic Alliance Skull Base Pathology Radboudumc/MUMC+, Nijmegen and Maastricht, The Netherlands; Department of Otolaryngology, Radboud University Medical Center, Nijmegen, The Netherlands; Dutch Academic Alliance Skull Base Pathology Radboudumc/MUMC+, Nijmegen and Maastricht, The Netherlands; Department of Otolaryngology, Radboud University Medical Center, Nijmegen, The Netherlands; Dutch Academic Alliance Skull Base Pathology Radboudumc/MUMC+, Nijmegen and Maastricht, The Netherlands; Gamma Knife Center, Department of Neurosurgery, Elisabeth-TweeSteden Hospital, Tilburg, The Netherlands; Department of Otolaryngology, Radboud University Medical Center, Nijmegen, The Netherlands; Department of Otolaryngology, Maastricht University Medical Center+, Maastricht, The Netherlands; Dutch Academic Alliance Skull Base Pathology Radboudumc/MUMC+, Nijmegen and Maastricht, The Netherlands

**Keywords:** Koos grade 4, observation, vestibular schwannoma, volumetric growth, wait-and-scan

## Abstract

**Background:**

Volumetric natural history studies specifically on large vestibular schwannomas (VSs), commonly classified as Koos grade 4, are lacking. The aim of the current study is to present the volumetric tumor evolution in sporadic Koos grade 4 VSs and possible predictors for tumor growth.

**Methods:**

Volumetric tumor measurements and tumor evolution patterns from serial MRI studies were analyzed from selected consecutive patients with Koos grade 4 VS undergoing initial wait-and-scan management between January 2001 and July 2020. The significant volumetric threshold was defined as a change in volume of ≥10%.

**Results:**

Among 215 tumors with a median size (IQR) of 2.7 cm^3^ (1.8–4.2), 147 tumors (68%) demonstrated growth and 75 tumors (35%) demonstrated shrinkage during follow-up. Growth-free survival rates (95% CI) at 1, 2, 5, and 10 years were 55% (48–61), 36% (29–42), 29% (23–36), and 28% (21–34), respectively and did not significantly differ in tumors> 20 mm (Chi-square = .40; *P*-value = .53). Four tumor evolution patterns (% of total) were observed: continued growth (60); initial growth then shrinkage (7); continued shrinkage (27); and stability (5). Good hearing (adjusted HR 2.21, 95% CI 1.48–3.30; *P* < .001) and peritumoral edema (adjusted HR 2.22, 95% CI 1.18–4.13; *P* = .01) at diagnosis were significantly associated with an increased likelihood of growth.

**Conclusions:**

Koos grade 4 VSs show a wide variety in size and growth. Due to variable growth patterns, an initial wait-and-scan strategy with short scan intervals may be an acceptable option in selected tumors, if no significant clinical symptoms of mass effect that warrant treatment are present.

**Key Points**

1. Growth prevalence in Koos grade 4 vestibular schwannomas seems comparable to smaller tumors.
2. More than 90% of tumor growth was observed within 2 years after diagnosis.
3. Approximately one-third of tumors demonstrated shrinkage.

**Importance of the Study**

There remains a global discrepancy in the management strategy of large VSs compressing the brain stem, commonly classified as Koos grade 4. Anticipated growth and its potential clinical impact are essential factors in clinical decision-making. However, proper evidence on the anticipated growth of these tumors is lacking. As wait-and-scan management generally is not recommended and therefore not typically done in daily practice, this data is inherently difficult to obtain. The striking observed shrinkage in approximately a third of the patients may put the conservative approach for Koos grade 4 VSs in a different light. Findings from this longitudinal volumetric study on Koos grade 4 tumors with long follow-up provide insightful new evidence on anticipated growth and contribute to the continuing debate on finding the optimal management strategy for large tumors.

Vestibular schwannomas (VSs) are benign tumors originating from the 8th cranial nerve and are the most common tumor of the cerebellopontine angle. Management strategies for VSs are either wait-and-scan (W&S), stereotactic radiosurgery (SRS), microsurgery (MS), or a combination of these methods. For newly diagnosed small- to medium-sized VS tumors, the conservative W&S management is increasingly becoming the preferred initial approach. When growth is observed, subsequent treatment with either stereotactic radiosurgery or microsurgery may be pursued.^1–3^ Alternatively, a continued W&S management can be chosen on individual basis.

However, for newly diagnosed large VSs, commonly classified as Koos grade 4, there remains a global discrepancy in the initial approach strategy.^4^ Due to the unpredictable nature of VS tumor growth and the potential clinical impact of mass effect, most centers opt for an active upfront approach, with microsurgery still being the first choice for Koos grade 4 tumors. Recently, the less invasive SRS strategy has gained more popularity and evidence as an alternative, safe, and effective treatment for selected large VSs.^2,4–7^ On the contrary, initial W&S management for Koos grade 4 VSs has been insufficiently investigated, as it is generally not recommended in recent guidelines and therefore not typically done in daily practice.^1–4^

Tumor size and anticipated growth are essential factors in clinical decision-making for VSs. In current natural history studies, larger tumor size at diagnosis is the most consistent predictor for future growth in literature.^1,8–11^ However, these previous natural history studies are mostly confined to linear measurements or smaller tumors. Volumetric natural history studies, specifically on large (Koos grade 4) vestibular schwannomas, are lacking. The existing knowledge gaps on the natural history of Koos grade 4 VSs may carry significant clinical implications in the management of these tumors. The primary aim of the current study was to assess the volumetric natural history and tumor evolution patterns in sporadic Koos grade 4 VSs and evaluate possible predictors for tumor growth.

## Methods

### Study Population

Institutional review board approval was obtained for this study. All newly diagnosed or referred patients with untreated unilateral sporadic VS, diagnosed between January 2001 and July 2020, were identified at the tertiary referral institute. The inclusion criteria were as follows: Koos grade 4, defined as VS tumors compressing the middle cerebellar peduncle (MCP); initial W&S approach; a minimum observation period of 1 year; and at least 2 serial MRI scans available for volumetric growth analysis. Patients requiring conversion to active treatment within 1 year, due to significant (≥ 2 mm) linear growth, were also included.

### Patient- and Tumor Characteristics

Baseline patient demographics (age at time of diagnosis and sex) and presenting symptomatology (hearing loss, tinnitus, vertigo, instability, facial paresis, and trigeminal dysfunction including facial numbness or facial pain) were collected from the institutional electronic medical records. The hearing function was scored using the Gardner–Robertson scale.^12^ Tumor-specific characteristics (cystic components, peritumoral edema, ventricular shift, and degree of brainstem compression) were obtained by reviewing the MR images and radiological reports. Presence of peritumoral edema was assessed on T2-weighted imaging. Presence of microcystic components is defined as having one or more small- to moderate intratumoral cysts and macrocystic components are defined as having large peripheral cysts compromising more than 50% of the total tumor volume.^13^ The degree of tumor middle cerebellar peduncle (MCP) compression was quantified by measuring the absolute and relative difference of the MCP width on the tumor side versus the width of the contralateral side in the axial plane, as measured in Hasawega et al.^14^ The patient- and tumor characteristics are summarized with medians and interquartile ranges (IQRs) or with frequency counts and percentages.

### Volumetric Follow-Up

Tumor volume measurements were manually or semi-automatically performed slice-by-slice with ITK-SNAP software (version 3.8.0) or GammaPlan (version 11.3, Elekta AB, Stockholm, Sweden) on postcontrast T1-weighted sequences or thin-slice heavily T2-weighted sequences, depending on the availability of MR images of sufficient quality (further specified in Supplementary Table S1).^15^ The minimum slice thickness required was 5 mm in postcontrast T1-weighted sequences or 2 mm in thin-slice heavily T2-weighted sequences. Either ITK-SNAP or GammaPlan was consistently used for all measurements within a patient to avoid any inter-software variability. Annotation was performed by the authors (S.S., S.C., P.L., and J.V.) with 3–21 years of experience in volumetric segmentation of VSs. Based on the results of an inter-observer variability study with the same annotators (S.C., et al., unpublished manuscript, 2023), the interrater correlation coefficient was 0.995, demonstrating excellent agreement between annotators.

The significant volumetric threshold for growth and shrinkage was defined as a longitudinal relative change in volume of ≥ 10%. Follow-up duration was calculated from the date of VS diagnosis to the date of the last follow-up.

### Statistical Analysis

Overall longitudinal tumor evolution patterns were assessed, visualized, and described. As most centers opt for upfront treatment for tumors larger than 15–20 mm extrameatal diameter,^1,16,17^ tumors with a maximum extrameatal diameter larger than 20 mm were separately evaluated. The growth rate was calculated using volume doubling time (VDT), as defined in Varughese et al.^18^ and using average relative growth per year by comparing volumes of the first and last (untreated) follow-up MRI scans. Growth-, intervention-, and shrinkage-free survival rates were estimated using the Kaplan–Meier method and the log-rank test for comparison.

Associations of baseline patient- and tumor characteristics with time to growth were evaluated, using uni- and multivariable Cox proportional hazard regression models and summarized with hazard ratios and 95% confidence intervals (CIs). Values for *P* < 0.05 were considered significant. Statistical analyses were performed with SPSS Statistics for Windows, Version 26 (IBM Corporation, Armonk, NY, USA).

## Results

### Patient Population

**Figure 1.**
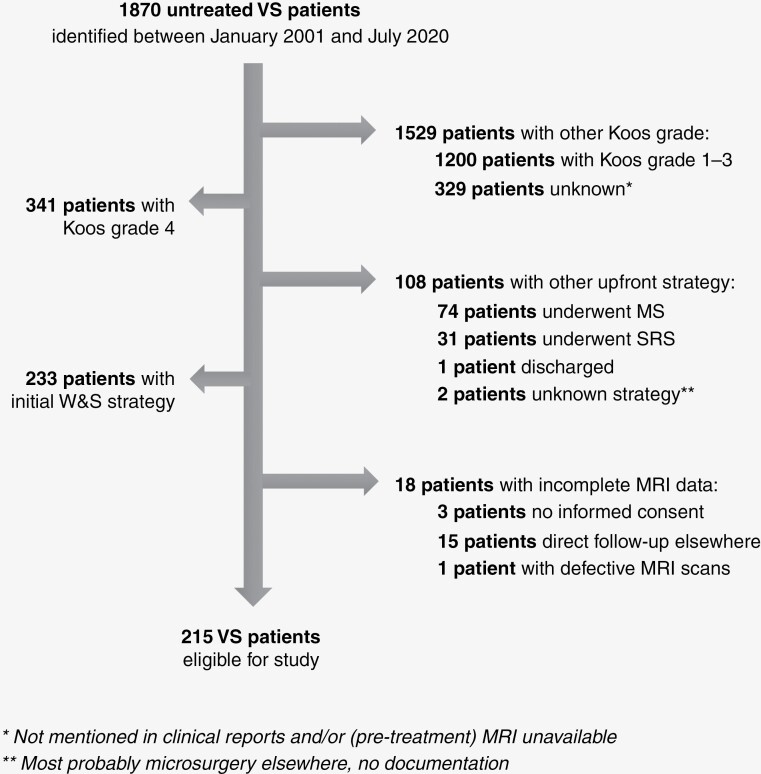
Flow diagram of the study population.

Between January 2001 and July 2020, 341 patients with untreated unilateral sporadic Koos grade 4 VS were identified. A W&S strategy was initiated for 233 patients (68.3%). Of these, 215 patients met the inclusion criteria (Figure 1). The median age at diagnosis was 58 years old (IQR 46–65). Concerning the presenting symptomatology at diagnosis, 127 patients (59%) had serviceable hearing (GR1 & 2), 25 patients (12%) had trigeminal dysfunction, and none had facial paresis. Median tumor size at diagnosis was 2.7 cm^3^ (IQR 1.8–4.2), 59 tumors (28%) had cystic components, and 24 tumors (11%) had peritumoral edema. Ultimately, 128 patients (60%) converted to active treatment, all due to observed radiographic progression: 114 patients underwent primary SRS; 11 patients underwent primary MS; and 3 patients underwent MS with adjuvant SRS. The median time to treatment was 16 months (IQR 11–27 months). The median time of follow-up among the patients who did not undergo treatment was 78 months (IQR 35–108). Patient- and tumor characteristics at the time of diagnosis and follow-up are summarized in Table 1.

**Table 1:** Summary of Patient- and Tumor Characteristics at Time of Diagnosis and Follow-Up^a^

Characteristic		All tumors *N* = 215	Tumors > 20 mm^b^ *N = *88
Age at diagnosis in years		58 (46–65)	57 (46–65)
Male gender		106 (50)	52 (59)
Gardner–Robertson scale	1	54 (25)	19 (22)
	2	73 (34)	25 (28)
	3	47 (22)	21 (24)
	4	17 (8)	10 (11)
	5	15 (7)	9 (10)
	Missing	8 (4)	4 (5)
Tinnitus		158 (74)	61 (69)
Instability		86 (40)	42 (48)
Vertigo		19 (9)	6 (7)
Trigeminal dysfunction		25 (12)	16 (18)
Facial numbness		25 (12)	15 (17)
Facial pain		5 (1)	5 (6)
Facial paresis		0 (0)	0 (0)
Volume in cm^3^		2.7 (1.8–4.2)	4.8 (3.6–6.2)
Diameter in mm ^b^		20 (17–23)	24 (22–27)
Number of follow-up MRIs		4 (3-6)	5 (3–6)
Cystic components		59 (28)	32 (36)
Microcystic		21 (10)	11 (13)
Macrocystic		38 (18)	21 (24)
Fourth ventricular shift		97 (46)	72 (82)
Peritumoral edema		24 (11)	22 (25)
Absolute MCP compression in mm		5 (4–7)	7 (6–9)
Relative MCP compression in %		33 (25–41)	43 (35–50)
Conversion to treatment		128 (60)	45 (51)
Stereotactic radiosurgery		114 (53)	32 (36)
Microsurgery		11 (5)	9 (10)
Microsurgery with adjuvant SRS		3 (1)	3 (3)
Time to treatment in months		16 (11–27)	16 (9–28)
Follow-up time in months		26 (15–72)	29 (13–71)
Follow-up time in months of non-growing tumors		79 (33–108)	68 (34–96)

^a^Summarized with median (IQR) or *n* (% of total).

^b^Maximum extrameatal diameter.

Abbreviation: SRS, stereotactic radiosurgery.

### Tumor Evolution

**Figure 2.**
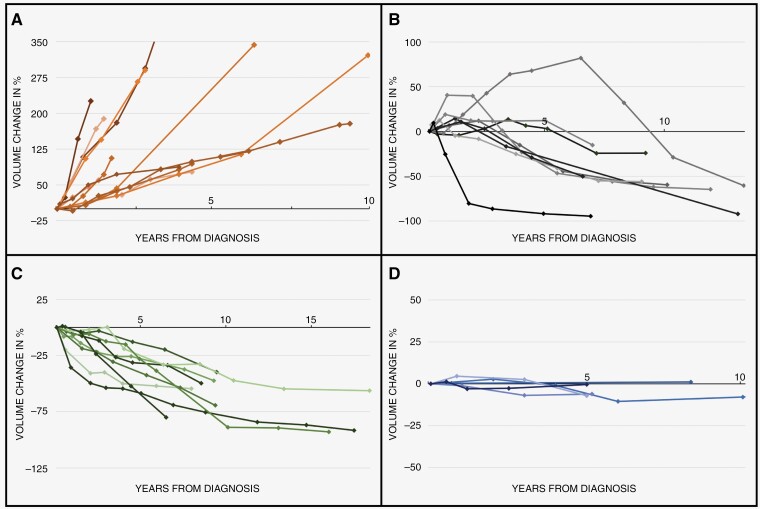
Illustrative line plots from selected representative tumors demonstrating the 4 tumor evolution patterns following diagnosis: (A) continued growth; (B) growth then shrinkage; (C) continued shrinkage; and (D) overall stability.

Of the initial 988 serial MRI studies performed, 957 MRI studies (96.9%) were available and of sufficient quality for volumetric tumor segmentation. Among the 215 tumors, growth and shrinkage were observed in 147 (68%) and 75 tumors (35%), respectively. Among the 88 tumors larger than 20 mm diameter, growth and shrinkage were observed in 53 (60%) and 39 (44%) tumors, respectively. Overall, 4 tumor evolution patterns were observed: (1) continued growth; (2) growth then shrinkage; (3) continued shrinkage; and (4) stability (Figure 2). A summary of the tumor evolution events and patterns is summarized in Table 2.

**Table 2.** Summary of Tumor Evolution Events and Tumor Evolution Patterns^a^

	All tumors *N* = 215	Tumors > 20 mm^b^ *N = *88
**Tumor evolution events**		
Growth	147 (68)	53 (60)
Time to growth in months	9 (6–15)	7 (6–16)
Shrinkage	75 (35)	39 (44)
Time to shrinkage in months	23 (15–54)	22 (11–47)
≥ 20% growth in volume	123 (58)	39 (44)
≥ 20% shrinkage in volume	60 (28)	30 (34)
**Tumor evolution patterns**		
A. Continued growth	130 (60)	45 (51)
Median follow-up time in months	16 (11–26)	15 (9–25)
VDT in months	20 (12–36)	24 (13–41)
Growth rate in % per year	56 (30–118)	42 (26–85)
B. Growth then shrinkage	14 (7)	5 (6)
Median follow-up time in months	104 (73–131)	77 (63–103)
Maximum volumetric increase in %	16 (13–30)	15 (12–23)
Time to subsequent shrinkage in months ^c^	31 (21–67)	21 (10–27)
Total subsequent volumetric shrinkage in % ^d^	55 (21–71)	55 (24–67)
C. Continued shrinkage	58 (27)	31 (35)
Median follow-up time in months	84 (55–112)	80 (59–107)
Total volumetric shrinkage in % ^e^	47 (22–60)	42 (22–67)
Shrinkage rate in % per year	7 (5–8)	7 (5–10)
D. Stability	10 (5)	4 (5)
Median follow-up time in months	31 (22–76)	23 (16–30)
E. Outliers	3 (1)	3 (3)

^a^Summarized with median (IQR) or *n* (% of total).

^b^Maximum extrameatal diameter.

^c^Following event of initial growth.

^d^At last follow-up compared to volume at maximum growth.

^e^At last follow-up compared to volume at diagnosis.

### Continued Growth

A total of 130 VS tumors (60.4%) showed a continued growth pattern with a median time to growth of 9 months (IQR 6–15). Of these, 123 patients converted to active treatment with a median time to treatment of 16 months (IQR 6–11). Two patients were lost to follow-up, while necessitating treatment due to observed linear growth. The median VDT is 20 months (IQR 12–36), and the median average relative growth is 56% (IQR 30–118) per year.

### Growth Then Shrinkage

Fourteen VS tumors (6.5%) showed a pattern of initial growth and then subsequent shrinkage. The initial growth of 11 tumors was not detected by linear measurements. Median time to initial growth was 11 months (IQR 6–17). Median time to subsequent shrinkage following the initial event of growth was 31 months (IQR 21–67). Two patients were converted to primary SRS, both due to presumed linear growth (in retrospect however not significant), after 74 months and 127 months follow-up, respectively.

### Continued Shrinkage

Fifty-eight VS tumors (25.4%) showed an overall pattern of continued shrinkage, with or without stabilization, with a median time to shrinkage of 21 months (IQR 12–45). The median total volumetric shrinkage at last follow-up, compared to the volume at diagnosis, was 47% (IQR 22–60), with a median average shrinkage rate of 6.7% (IQR 4.7–8.4%) per year. One patient, with an observed total shrinkage of 12%, was converted to primary SRS after 12 months of follow-up due to presumed clinical tumor growth based on linear measurements (in retrospect however not significant).

### Stability

Ten VS tumors (4.7%) remained stable during follow-up. The median follow-up time was 31 months (IQR 22–76). None were converted to treatment during follow-up.

### Outliers

Three VS tumors (1.4%) showed dissimilar growth patterns. Two tumors had cystic components and both demonstrated initial solid tumor shrinkage, with a sudden progression of the cystic components during follow-up, thereby necessitating conversion to active treatment. One patient received primary SRS and the other patient received primary MS. The third VS tumor demonstrated a pattern of initial growth, followed by subsequent shrinkage caused by regression of the intratumoral hemorrhage, and subsequent-subsequent growth. This patient was initially lost to follow-up during the COVID pandemic and returned with mass-effect-related complications. Due to old age and comorbidities, the patient could not be treated and died. Further details are presented in Supplementary Figure S1 and Supplementary Table S2.

### Survival analyses

**Figure 3.**
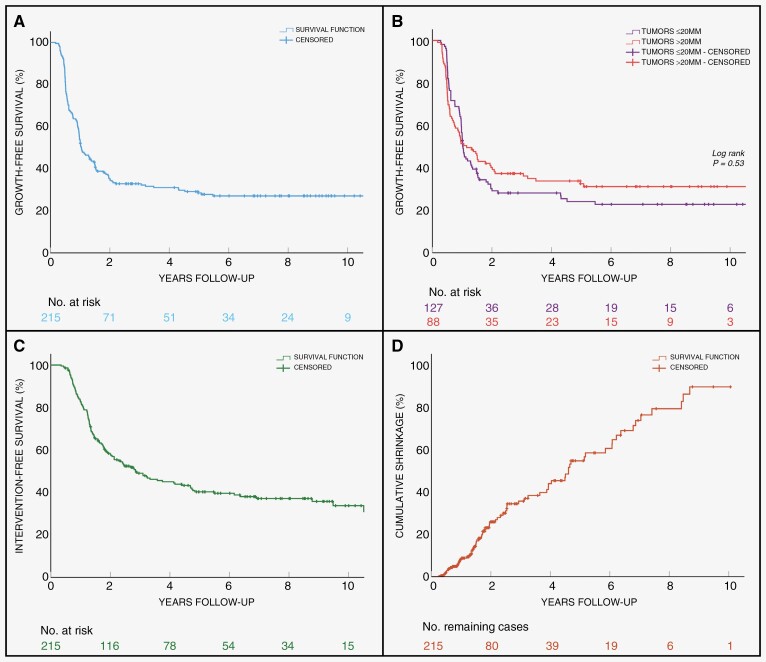
Kaplan–Meier survival curves of: (A) growth-free survival rates; (B) growth-free survival rates of tumors > 20 mm maximum extrameatal diameter versus tumors ≤ 20 mm diameter maximum extrameatal diameter; (C) intervention-free survival rates; and (D) cumulative shrinkage rates.

Growth-free survival rates (95% CI; numbers still at risk) at 1, 2, 5, and 10 years were 55% (48–61; 114), 36% (29–42; 71), 29% (23–36; 42), and 28% (21–34; 9), respectively (Figure 3A). Growth-free survival rates did not significantly differ (Chi-square = .302; *P*-value = .58) in tumors larger than 20 mm compared to tumors of 20 mm and smaller (Figure 3B). Intervention-free survival rates at 1, 5, and 10 years were 82% (77–88; 173), 41% (34–48; 63), and 34% (26–42; 14), respectively (Figure 3C). Cumulative shrinkage rates at 1, 2, and 5 years were 8% (4–13; 159), 25% (19–34; 80), and 55% (48–70; 25), respectively (Figure 3D).

### Cox Proportional Hazards for Growth

Uni- and multivariable associations of baseline patient- and tumor-specific features for risk of growth are displayed in Table 3. Only hearing class and peritumoral edema had a significant association with growth. Good hearing (HR 2.21; 95% CI:1.48–3.30) and peritumoral edema (HR 2.22; 95% CI: 1.18–4.13) both showed an adjusted 2.2-fold increased likelihood of growth. Interaction between size and age was additionally assessed and showed no significance for growth.

**Table 3.** Uni- and Multivariable Analyses of Features at Diagnosis With Risk for Growth.

		Growth			
Features		HR (95% CI)	*P*-value	Adjusted HR (95% CI)^c^	*P*-value
Age at diagnosis in years^a^	24–47	(Ref)	N/A	(Ref)	N/A
	47–58	1.11 (0.72–1.74)	.62	0.90 (0.55–1.46)	.67
	58–65	0.83 (0.52–1.32)	.42	0.67 (0.40–1.11)	.12
	65–88	0.92 (0.58–1.46)	.92	0.75 (0.43–1.29)	.29
Volume in cm^3^^a^	0.7–1.8	(Ref)	N/A	(Ref)	N/A
	1.8–2.7	0.87 (0.56–1.36)	.55	0.94 (0.57–1.54)	.80
	2.7–4.2	0.97 (0.62–1.51)	.89	0.89 (0.48–1.63)	.71
	4.2–20.3	0.80 (0.50–1.30)	.37	0.80 (0.40–1.58)	.52
Gardner–Robertson scale	1	(Ref)	N/A	(Ref)	N/A
	2	**0.49 (0.32–0.74)**	**<.001**	**0.40 (0.25–0.63)**	**<.001**
	3	**0.50 (0.31–0.79)**	**.003**	**0.44 (0.27–0.73)**	**.002**
	4	0.83 (0.44–1.56)	.55	0.70 (0.36–1.37)	.30
	5	0.68 (0.34–1.35)	.27	0.70 (0.33-–.47)	.35
Serviceable hearing (GR1&2)		1.11 (0.79–1.56)	.56		
Good hearing (GR1)		**1.88 (1.31–2.68)**	** < .001**	**2.21 (1.48–3.30)**	** < .001**
Tinnitus		1.09 (0.76–1.56)	.65		
Instability		1.12 (0.81–1.56)	.50		
Vertigo		1.01 (0.57–1.79)	.96		
Trigeminal dysfunction^b^		0.98 (0.59–1.63)	.79		
Cystic components		0.73 (0.50–1.07)	.10		
Ventricular shift		0.98 (0.71–1.36)	.90		
Peritumoral edema		**1.65 (1.02–2.68)**	**.04**	**2.22 (1.18–4.13)**	**.01**
Absolute MCP compression^a^	2–4 mm	(Ref)	N/A		
	5 mm	0.99 (0.62–1.56)	.95		
	6–7 mm	1.06 (0.71–1.58)	.79		
	8–18 mm	1.05 (0.66–1.67)	.83		
Relative MCP compression^a^	11–24%	(Ref)	N/A		
	25–32%	1.11 (0.71–1.73)	.66		
	33–41%	1.02 (0.65–1.62)	.92		
	42–82%	1.14 (0.72–1.81)	.57		

^a^Categorized in quartiles due to a non-linear relationship with outcome

^b^Trigeminal dysfunction includes facial numbness and/or facial pain.

^c^Multivariable analyses were only performed for age, size, and significant associations (marked in bold text) in univariable analyses. HR is adjusted for age, size, tinnitus, instability, vertigo, hearing class, trigeminal dysfunction, cystic components, ventricular shift, and peritumoral edema.

Abbreviations: MCP, middle cerebellar peduncle; GR, Gardner–Robertson hearing scale.

## Discussion

In the current study, 68% of all tumors demonstrated significant growth during follow-up, and 60% of all tumors demonstrated a continued growth pattern. Both rates are comparable to volumetric reports on smaller VSs, ranging from 42% to 79%. This is particularly interesting considering the larger tumor size, lower volumetric change thresholds, and longer median follow-up in this study.^10,11,19–21^ Furthermore, in the Cox hazard regression and survival analyses, larger tumor size was not significantly associated with an increased likelihood for growth. This is a contradicting observation, as larger size appears to be the most consistent predictor for growth or continued growth in literature. However, these studies are mostly confined to linear growth or smaller tumors, where median tumor volumes range from 0.1 to 0.6 cm^3^ and median maximum extrameatal diameters are around 10 mm.^10,11,19–21^ In this cohort, the median tumor volume was 2.7 cm^3^ with a median maximum extrameatal diameter of 20 mm. This major difference indicates the inapplicability of extrapolating findings in previous reports to larger VSs. The observations in this study on Koos grade 4 tumors suggest that the anticipated growth prevalence seems more comparable to smaller VSs than initially thought.

Most growth was observed within the first few years of follow-up. Two-thirds of growth was observed within 1 year and 90% within 2 years follow-up. The last growth event was observed at 5.5 years after diagnosis. This suggests that when opting for an initial W&S approach, there will be a high degree of certainty that the tumor will remain non-growing and will not necessitate conversion to active management at merely 2 years of volumetric follow-up. When comparing the median time to growth and the median time to treatment in the overall growing tumors, there is an interval of approximately 7 months. Whilst not all relevant, it does indicate some preventable treatment delays. Especially in large tumors this interval could be of clinical significance, particularly when tumors are fast growing and may surpass the window of opportunity for the less invasive SRS. It is well known that VS growth rates are heterogenic,^11,18,21,22^ this study also demonstrates a wide range in growth rates. Both results on VDT and relative growth rate per year show that approximately a quarter of tumors at least double in volume within 1-year follow-up. Purely based on the growth rate, this indicates the necessity for close monitoring with short scan intervals during these first years of follow-up. In current clinical practice, linear measurements are still used for detecting growth. It has already been reported repeatedly that volumetric measurements are more sensitive, resulting in earlier growth detection.^11,20,23–26^ However, manual or semi-automatic volumetric measurements are time-intensive and therefore often not feasible in clinical practice to monitor all VSs volumetrically, not until reliable fully automatic segmentation tools are available. For smaller VSs, linear monitoring is usually clinically sufficient, especially when growth does not necessarily have clinical consequences in treatment management.^17,27,28^ However, in large VS tumors, observed growth does usually have direct clinical consequences. Thus, when opting for conservative management, volumetric monitoring should be considered as standard-of-care in clinical practice for large VSs.

Recent studies have observed already a variety of evolution patterns in VSs, for example, regression, initial growth followed by a period of tumor stability, or even oscillation.^10,29–32^ In this study, various tumor evolution patterns could also be distinguished, and tumors could systematically be categorized into 4 different patterns: continued growth; initial growth followed by subsequent shrinkage; continued shrinkage; and stability. Other studies have observed similar patterns to these.^30–32^ However, the distribution of the observed patterns in this study was striking. A clear minority (5%) of the tumors remained stable during follow-up. Excluding the outliers, approximately a third (34%) of all tumors demonstrated significant shrinkage. The 7-fold difference of shrinkage versus stability has not been reported before, where cases of shrinkage in previous volumetric reports range from 1% to 14%. This intriguing result could be explained by several factors. First, the median follow-up time is longer compared to other reports, that is, a median MRI surveillance time of 2.2 years (IQR 1.3–6.0) for the entire cohort and 6.6 years (IQR: 2.8–9.0) among patients who did not undergo treatment. The Kaplan–Meier curves showed shrinkage events were spread over time, almost 50% and 20% of the cumulative shrinkage events were only observed after 2 and 5 years of follow-up, respectively. Thus, having a longer follow-up time, more shrinkage events could be witnessed. Another possible source of the higher shrinkage rate may be related to the larger tumor size in this cohort. Tikka et al.^32^ observed that larger tumor size was significantly associated with shrinkage. It could be thought that the lower thresholds of 10% may also have an additional influence. However, when holding the frequently used threshold of 20%, we still observed 28% shrinkage in tumors. Therefore, the observed significant degree of shrinkage is undeniable and could have significant implications on clinical decision-making. In current practice, clinical decision-making mainly focuses on anticipating growth. With this amount of shrinkage, predicting shrinkage would also be of interest. As this surpasses the initial scope of this study, further analysis on shrinkage is not included but certainly will be pursued in future research.

In Cox regression analyses, good hearing and the presence of peritumoral edema at diagnosis both had a significantly increased likelihood for growth. When adjusting for the other patient- and VS-related variables, the association strengthens and increases to more than a 2-fold likelihood for both variables. Good hearing was perhaps a slightly contradictive association, which has not been reported before. This may be due to the nature of this unique cohort of large VSs. A hypothesis could be that when following the lifespan of a VS, growing tumors have not been present for that long and therefore relatively unaffected hearing, compared to the stabilized or regressing tumors that have already been compressing the vestibulocochlear nerve for years. Important to note that this is a cross-sectional measurement at diagnosis and does not take the duration and progression of symptoms into account, which also may have additional predictive value, as previously reported in Hentschel et al.^8^ Peritumoral edema was another finding associated with growth. Peritumoral edema does not always occur and can even be absent despite a large tumor size with severe MCP compression. This may suggest that tumor growth may influence the emergence of edema. Age and tumor size are important clinical factors within clinical decision-making, however, both failed to demonstrate any association or interaction for future growth.

The definition of significant growth and shrinkage is an essential but complex factor in a study’s methodology, with a significant impact on growth outcome. In an interobserver variability study on volumetric VS segmentation (S.C., et al., unpublished manuscript, 2023), we observed that the smallest detectable change or limits of agreement decreases with larger tumor volume, as observed by Van den Langenberg et al.^33^ Based on these results and considering the larger tumor size in this cohort, we predefined significant volumetric change as 10%. The more frequently used threshold of 20% would clearly encompass a substantial number of false stability, other studies have expressed similar concern for larger tumors including the potential clinical risk of delay.^11^ Only 3 outliers showed dissimilar patterns in this study, which were clinically explicable due to sudden cystic changes or an intratumoral hemorrhage. In conclusion, with this precise threshold definition combined with long follow-up, the results give an accurate representation on VS tumor evolution in time.

The optimal management strategy for large vestibular schwannomas remains to be a continuing global debate. In current guidelines, an initial conservative approach for newly diagnosed Koos grade 4 tumors is not considered as an acceptable option, despite the wide range in tumor size and clinical profile Koos grade 4 tumors represent.^2,3^ In this study, the striking observed shrinkage in approximately a third of the patients puts the conservative approach for selected Koos grade 4 VSs, that is, cases without clinical symptoms of mass effect, in a different light. It is important to note that this study purely analyzed volumetric changes in tumors, and merely provides evidence on anticipated growth in large VSs. To properly assess the limits and clinical implications of W&S versus upfront treatment in specifically large VSs, other clinical outcome measures should be evaluated prospectively, for example, hearing function, neurological status, and quality of life.

There are limitations in this study. First of all, as a tertiary referral institute, there is an obvious degree of referral bias. However, in the Netherlands all newly diagnosed VSs should be referred to a tertiary skullbase institute, thus the referral bias being mainly of geographical origin. Next, the foremost limitation consists of a selection bias in clinical decision-making for managing Koos grade 4 VS patients conservatively. This has implications for several relevant patient characteristics in this cohort. First, the median size of 2.7 cm^3^, or 20 mm extrameatal diameter, is small when compared to the median VS sizes with upfront therapy in our center (initial SRS: 7.6 cm^3^; initial MS: 12.4 cm^3^; see Supplementary Table S3) and to other Koos grade 4 SRS and MS studies.^6,7,34,35^ This also proves how the definition of Koos grade 4 VSs consists of a very wide range of tumor sizes and could be a misleading classification.^34,36,37^ While most centers opt for upfront treatment for tumors larger than 15–20 mm extrameatal diameter,^1,16,17^ our center generally holds a higher threshold of 25–30 mm, depending on tumor shape and risk of missing the window of opportunity for SRS. Other reasons favoring upfront intervention in multidisciplinary clinical decision-making consist of presence of neurological symptoms of mass effect; hydrocephalus; extensive peritumoral edema; severe MCP compression; young age; and facial nerve paresis. Other than a right-skewed size distribution, this selection results in notable observations in our cohort characteristics: no patients with clinical symptoms of mass effect, hydrocephalus, or facial paresis; a lower prevalence of peritumoral edema and trigeminal dysfunction; and slightly older patients compared to the upfront Koos grade 4 treatment cohort in our center (Supplementary Table S3). Nevertheless, the median age of 58 years old is comparable to other natural history populations. Another limitation is that the overall growth pattern may be overestimated since nearly all tumors with observed clinical growth were converted to an active treatment regimen. There is therefore no knowledge of tumor evolution thereafter, which may underestimate the rate of subsequent shrinkage after the initial growth of tumors. However, for large Koos grade 4 tumors, this knowledge is practically unfeasible to obtain in daily practice: when growth is observed, active treatment should be considered in order to prevent an increased risk for complications for active treatment due to larger tumor size. Hence, despite these limitations, this study gives a realistic insight into the natural course of large VSs and for VSs in general.

In conclusion, this large volumetric study provides new evidence on anticipated growth in sporadic Koos grade 4 VSs, contributing to the continuing debate on finding the optimal management strategy for these tumors. Koos grade 4 VSs include a wide range in size and tumor evolution. In approximately a third of the patients, relevant shrinkage is observed. A W&S strategy with short scan intervals may therefore be an acceptable option in selected cases if no significant clinical symptoms of mass effect that warrant treatment are present. Further research on prospective matched-cohort research on clinical outcomes and implications should be pursued.

## Supplementary Material

vdad144_suppl_Supplementary_Tables_S1

vdad144_suppl_Supplementary_Figures_S1

vdad144_suppl_Supplementary_Tables_S2

vdad144_suppl_Supplementary_Tables_S3

## Data Availability

The data that support the findings of this study are not publicly available due to restrictions applying to the availability of these data. Data are, however, available from the corresponding author upon reasonable request and are located in controlled access data storage at the Radboud University Medical Center, Nijmegen, the Netherlands.
