# Supplementary material for: Wait-and-scan management in sporadic Koos grade 4 vestibular schwannomas: A longitudinal volumetric study
Source: Neurooncol Adv. 2023 Nov 3;6(1):vdad144. doi: 10.1093/noajnl/vdad144 (PMC10771273; doi:10.1093/noajnl/vdad144)
Supplement: vdad144_suppl_Supplementary_Tables_S1 [file vdad144_suppl_supplementary_tables_s1.docx]

**Supplemental Table S1. Minimum MRI requirements for analysis**

|  | **T1-contrast enhanced** | **Heavily T2-weighted** |
| --- | --- | --- |
| Maximum slice thickness | 5mm | 2mm |
| Minimum resolution | 256x256 | 512x512 |
| Minimum slices of tumor to segment | 5 | 5 |
