# Supplementary material for: Wait-and-scan management in sporadic Koos grade 4 vestibular schwannomas: A longitudinal volumetric study
Source: Neurooncol Adv. 2023 Nov 3;6(1):vdad144. doi: 10.1093/noajnl/vdad144 (PMC10771273; doi:10.1093/noajnl/vdad144)
Supplement: vdad144_suppl_Supplementary_Figures_S1 [file vdad144_suppl_supplementary_figures_s1.docx]

**Supplemental Figure S1 Legend**

Line plots demonstrating the tumor evolution following diagnosis of the three outliers: (A) Two cystic tumors with initial tumor regression of the solid component, during follow-up sudden cystic changes with progression of the cystic components; (B) solid tumor with initial growth due to an intralesional hemorrhage, followed by subsequent shrinkage due to regression of the hemorrhage, followed by subsequent-subsequent solid tumor growth.
