# Supplementary material for: Wait-and-scan management in sporadic Koos grade 4 vestibular schwannomas: A longitudinal volumetric study
Source: Neurooncol Adv. 2023 Nov 3;6(1):vdad144. doi: 10.1093/noajnl/vdad144 (PMC10771273; doi:10.1093/noajnl/vdad144)
Supplement: vdad144_suppl_Supplementary_Tables_S2 [file vdad144_suppl_supplementary_tables_s2.docx]

**Supplemental Table S2.** Patient- and tumor characteristics of outliers.

|  | **Outlier 1** | **Outlier 2** | **Outlier 3** |
| --- | --- | --- | --- |
| **Age at diagnosis** | 50 years old | 47 years old | 88 years old |
| **Volume at diagnosis** | 3.5cm^3^ | 6.3cm^3^ | 4.1cm^3^ |
| **Extrameatal maximum diameter** | 22mm | 26mm | 27mm |
| **Gardner-Robertson scale at diagnosis** | 1: Good | 2: Serviceable | 5: None |
| **Tinnitus at diagnosis** | Yes | No | No |
| **Vertigo at diagnosis** | No | No | Yes |
| **Instability at diagnosis** | Yes | No | Yes |
| **Trigeminal dysfunction at diagnosis** | No | No | No |
| **Peritumoral edema**  **At diagnosis** | No | No | Yes |
| **Cystic components**  **At diagnosis** | Microcystic components | Macrocystic  components | Solid |
| **Conversion to active treatment** | Primary SRS, single session Gamma Knife.  At 27 months follow-up. | Microsurgery subtotal resection retrosigmoid approach  At 39 months follow-up | No, lost to follow-up due to COVID pandemic. Returned 4 years later with mass-effect related complications. Due comorbidities unfit for surgery. |
| **Last follow-up** | Last follow-up 2.7 years post-SRS.  Tumor regression. | Last follow-up 3 years post-surgery.  Tumor remnant stable. | Died. |
| **Facial nerve function**  **Post-treatment** | House-Brackmann 1 | House-Brackmann 1 | N/A |
| **Trigeminal dysfunction**  **Post-treatment** | Numbness mouth corner | None | N/A |
| Abbreviation: SRS, stereotactic radiosurgery; | | | |
