# Supplementary material for: Wait-and-scan management in sporadic Koos grade 4 vestibular schwannomas: A longitudinal volumetric study
Source: Neurooncol Adv. 2023 Nov 3;6(1):vdad144. doi: 10.1093/noajnl/vdad144 (PMC10771273; doi:10.1093/noajnl/vdad144)
Supplement: vdad144_suppl_Supplementary_Tables_S3 [file vdad144_suppl_supplementary_tables_s3.docx]

**Supplemental Table S3.** Comparison patient characteristics between different initial regimen strategies^a^

| **Characteristic** | | **Initial W&S** | | **Initial SRS**  ***N = 31*** | **Initial MS**  ***N = 74*** | ***P*-value^b^** |
| --- | --- | --- | --- | --- | --- | --- |
|  |  | ***Total***  ***N = 215*** | ***>20mm***  ***N=88*** |  |  |  |
| Age at diagnosis in years | | 58 (46-65) | 57 (46-65) | 50 (43-53) | 52 (40-63) | .001 |
| Gardner-Robertson | 1  2  3  4  5  Missing | 54 (25)  73 (34)  47 (22)  17 (8)  15 (7)  8 (4) | 19 (22)  25 (28)  21 (24)  10 (11)  9 (10)  4 (5) | 6 (19)  11 (35)  3 (10)  3 (10)  4 (13)  4 (13) | 17 (22)  14 (19)  18 (24)  10 (14)  10 (14)  5 (7) | .06 |
| House-Brackmann grade | 1  2  3  4  5  6 | 214 (100)  0 (0)  0 (0)  0 (0)  0 (0)  0 (0) | 88 (0)  0 (0)  0 (0)  0 (0)  0 (0)  0 (0) | 31 (100)  0 (0)  0 (0)  0 (0)  0 (0)  0 (0) | 68 (92)  4 (6)  2 (2)  0 (0)  0 (0)  0 (0) | .003 |
| Trigeminal dysfunction | | 26 (12) | 16 (18) | 3 (10) | 21 (29) | .002 |
| Cerebellar dysfunction | | 0 (0) | 0 (0) | 0 (0) | 9 (12) | <.001 |
| Volume in cm^3^ | | 2.7 (1.8-4.2) | 4.8 (3.6-6.2) | 7.6 (5.7-10.9) | 12.4 (7.8-17.5) | <.001^c^ |
| Maximum extrameatal  diameter in mm | | 20 (17-23) | 24 (22-27) | 28 (25-32) | 32 (27-38) | <.001 |
| Peritumoral edema | | 24 (11) | 22 (25) | 10 (29) | 35 (47) | <.001 |
| Hydrocephalus | | 0 (0) | 0 (0) | 1(3) | 10 (14) | <.001 |
| *^a^ Summarized with median (IQR) or n (%)*  *^b^ One-way ANOVA analysis with Bonferroni correction for continuous variables and Chi-square analysis for categorical or dichotomous variables between total initial W&S, initial SRS and initial MS cohort.*  *^c^ log-transformed* | | | | | | |
